# Melatonin ameliorates paclitaxel‐induced mice spermatogenesis and fertility defects

**DOI:** 10.1111/jcmm.17177

**Published:** 2022-01-09

**Authors:** ZhiXin Wang, Zi Teng, ZeLin Wang, Zhan Song, Peng Zhu, Ning Li, YuSheng Zhang, XueXia Liu, FuJun Liu

**Affiliations:** ^1^ Shandong Stem Cell Engineering Technology Research Center Affiliated Yantai Yuhuangding Hospital of Qingdao University Yantai China

**Keywords:** melatonin, paclitaxel, sperm quality, spermatogenesis, testis

## Abstract

Chemotherapeutic drug of paclitaxel (PTX) has been shown to cause reproductive toxicity thus affecting male fertility, but its underlying molecular basis is unclear. Melatonin (MLT) can mitigate the reproductive damage caused by certain chemotherapy drugs. In this study, we aimed to identify impact of PTX on the main biological processes and protective effect of MLT on reproductive damage caused by PTX. Mice exposed to PTX mainly impaired spermatogenesis, such as decreased sperm counts, reduced sperm motility and increased abnormal sperm. Decreased expressions of germ cell proliferation‐associated protein PCNA and meiosis‐related protein SYCP3 induced by PTX were determined by Western blot, while MLT ameliorated this effect and increased the expressions of PCNA, SYCP3, DMC1, STRA8 and fertility‐related protein of HSPA2, resulting in significantly improved spermatogenesis and sperm quality levels. In vitro fertilization experiment showed that PTX significantly decreased blastocyst formation rates, which can be improved by MLT administration, but not two‐cell development rates. Taken together, this work demonstrated PTX can adversely affect germ cell proliferation and meiosis, which ultimately influence sperm quality and male fertility, and highlighted the protective ability of MLT on ameliorating the side effects of PTX, especially on sperm quality. The results provide information to further the study on the molecular mechanism of PTX's effects on male reproduction and the protective mechanism of MLT.

## 1 INTRODUCTION

As an important mean for cancer treatment, chemotherapy can increase the survival time of cancer patients. However, chemotherapy also has many side effects that may impact the life quality after treatment. The effect on fertility is a topic of great concern, especially for male fertility.^1^, ^2^ Reproductive side effects on male individuals include the damage to the testicular germinal epithelium and the sperm microenvironment, and the disorder of spermatogenesis and sperm maturation. The extents of the effects depend on patient age, as well as type, dosage and duration of drug.^3^ Paclitaxel (PTX), a diterpenoid compound, was first isolated from the bark of Undulata alba, and its chemical structure was determined by Wani et al.^4^ Now, PTX has become a conventional chemotherapy drug for cancer treatment, such as ovarian, uterine, breast, lung, oesophageal, prostate and rectal cancers.^5^ The anticancer mechanism of PTX is mainly through inhibiting the depolymerization of microtubules, which terminates the mitosis of tumour cells and causes the apoptosis of tumour cells, finally leading to the death of tumour cells.^6^

Although the concrete stage is not clear, the reproductive side effects of PTX are also evident, particularly in the testes. A single dose of PTX can significantly induce rat testicular changes with decreased sperm motility.^7^ PTX can lead to abnormal spermatogenesis, decreased sperm motility and sperm counts, increased numbers of abnormal sperm and apoptosis. Excessive oxidative stress is considered to be one of the important factors leading to these injuries.^8^ Overstock of ROS significantly impairs spermatogenesis and causes apoptosis in spermatogonia,^9^ while antioxidants may slow down oxidative stress levels and are used to diminish the detrimental effects caused by chemotherapy drugs.

Melatonin (MLT) is a neuroendocrine hormone secreted by the pineal gland.^10^ It was first purified in 1958 from the pineal gland extract of cattle.^11^ MLT performs a variety of physiological functions, including the regulation of biological rhythm, stability of gonadal function and maintenance of normal activities of the nervous and mental system, and it is also related to immune regulation, antioxidant and anti‐tumour activities.^12^ MLT is easy to cross the blood‐brain barrier and enters each brain region; it can effectively clear ROS and protect the oxidative damage of central nervous system and up‐regulate the expression of antioxidant enzymes in brain; so, it has a strong neuro‐protective effect.^13^ MLT also has a protective effect on liver, lung, kidney and cardio‐toxicity.^14^, ^15^ Because of its anti‐mitotic activity, immune‐modulatory activity, strong free radical scavenging ability and mitochondrial oxidative phosphoric acid uncoupling ability, MLT combined with chemotherapy or radiotherapy can significantly improve the survival rate of patients.

MLT also has potential effects on protecting male reproduction, while its protective role in reproductive damage caused by chemotherapeutic drugs has been less studied. In this work, the effects of PTX on key biological processes related to male fertility and the protective effects of MLT on reproductive damage caused by PTX were investigated in two aspects of spermatogenesis and sperm quality. PTX led to obvious changes in testicular structure, significantly affected germ cell proliferation, meiosis and subsequent sperm function. MLT, an efficient antioxidant, could protect sperm function from oxidative damage caused by PTX, resulting in the male fertility conservation. Our observations provide information to further understand the effects of PTX on spermatogenesis and sperm quality and to explore the fertility preservation effect of MLT on chemotherapy‐treated patients.

## 2 MATERIALS AND METHODS

### 2.1 Animals

Male ICR mice (8 weeks old, 29–32 g, Beijing Vital River Laboratory Animal Technology Co., Ltd.) were housed under a 12/12 light/dark cycle at constant room temperature (23 ± 1℃) with free access to food and water before initiation of the experiment. All procedures for animal care and use were carried out by following the guidelines for the care and use of laboratory animals. All efforts were made to reduce the number of animals used and bodily suffering during the experiment. This study was approved by the Medical Ethics Committee of Yantai Yuhuangding Hospital (No. 2020–155).

### 2.2 Construction of PTX‐induced mouse model

The experiments consisted two sections of PTX‐treated mouse model construction and MLT protection analysis. First of all, 60 mice were randomly divided into two groups (30 mice in control group and 30 mice in treatment group), and 5 mice were killed on Days 3, 5, 7, 14, 21 and 28 after treatment respectively. The collected samples including testis and epididymal sperm were used to detect sperm parameters and related changes in spermatogenesis. Then, the mouse model after 14 day treatment with PTX was selected to conduct the further study. The MLT protection experiments were randomly classified into four groups consisting of 10 animals each and treated as follows: Group 1 (Control group): Mice injected intraperitoneally (i.p.) with physiological saline once a week for 2 weeks were served as negative control. Group 2 (PTX treatment group): Mice were treated i.p. with PTX (10 mg/kg body weight/day) once. Group 3 (MLT): Mice were given continual i.p. injection of MLT (10 mg/kg body weight) for 2 weeks. Group 4 (MLT+PTX): Mice were injected i.p. with PTX (10 mg/kg body weight/day) once followed by 2 consecutive weeks i.p. injection of MLT (10 mg/kg body weight). Mice were taken for analysis at the 2nd week after PTX and MLT administration. Mice were euthanized by cervical dislocation, with one testis fixed in Bouin's fluid for histological evaluation and one frozen in liquid nitrogen and stored at −80℃ for protein extraction. The relative weight of the testis was expressed as g/g with body weight.

Mice blood was collected in anaesthetized mice to measure testosterone levels. Whole blood was centrifuged at 4℃ at 3000 *g* for 10 min to collect the serum. Serum testosterone was quantitatively detected by liquid chromatography‐tandem mass spectrometry.

Mice caudal epididymal sperm were harvested for analysis of sperm parameters and sperm function. Briefly, caudal epididymides were separated, cut into several segments and incubated at 35℃ for 5 min in PBS buffer. Sperm parameters were measured by using the Computer Aided Semen Analysis system (CASA) System (Medealab^™^). Sperm vitality was examined using eosin‐nigrosin staining method. Live spermatozoa have white heads and dead spermatozoa have heads that were stained red or dark pink. The average percentage of vital spermatoza were reported as vitality index.

The morphology of mice testes was observed by haematoxylin and eosin (HE) staining. Tissue specimens were sequentially fixed with Bouin's fluid, embedded and sectioned. De‐waxing was performed alternately by xylene and alcohol; then, the sections were stained in haematoxylin solution for 1 min, rinsed in running water and stained in working eosin solution for 1 min. Sections were dehydrated and observed under a light microscope (DM LB2, Leica). Score evaluation according to Bergamann and Kliesch was performed to describe spermatogenesis qualitatively.^16^

### 2.3 Western blotting

Testis proteins were lysed in RIPA lysis buffer (50 mM Tris‐HCl pH 7.4 150 mM NaCl, with protease inhibitor cocktail). 50 μg proteins from each sample were loaded and separated by 12% gels for sodium dodecyl sulphate‐polyacrylamide gel electrophoresis (SDS)‐PAGE. The gels were transferred to polyvinylidene difluoride membranes at 100 v for 1 h, blocked with 5% (w/v) skimmed milk for 1 h at room temperature (RT) and incubated with primary antibodies at 4℃ overnight with gentle agitation (Dilutions for various antibodies were indicated in supplementary Table 1). The membranes were washed with 0.5% (v/v) Tween‐20 in tris‐buffered saline (TBS) three times and incubated with horseradish peroxidase (HRP)‐conjugated anti‐IgG for 1 h at RT. The immune‐reactive signals were detected by an enhanced chemiluminescence (ECL) kit (Amersham Life Science). The expression band of each protein was recorded and analysed with ImageJ software. The average grey values of each band were normalized to that of ACTB. Western blotting experiments were performed at least three times with similar results.

### 2.4 Immunohistochemistry (IHC)

After de‐waxing and dehydration, antigen retrieving was conducted in a microwave oven for 20 min, and endogenous peroxidases were eliminated by incubation with 3% (v/v) H_2_O_2_ for 10 min. After blocked with 3% (w/v) bovine serum albumin (BSA) in tris‐buffered saline (TBS) at RT for 1 h, sections were then incubated with the primary antibody overnight at 4℃ (Supplementary Table 1). After washed three times with TBS, the sections were incubated with horseradish peroxidase‐conjugated IgG (Zhong‐Shan Biotechnology) at a final dilution of 1:400 for 1 h at 37℃. A 3, 3’‐diaminobenzidine (DAB) kit (Zhong‐Shan Biotechnology) was used to visualize peroxidase activity at the binding sites, and haematoxylin was used to counterstain the sections. The sections were dehydrated and mounted for bright‐field microscopy (DM LB2, Leica). Pre‐immune IgG was used as negative control.

The immune‐stained sections were captured by optical microscope, and the positive signals were analysed with image analysis software (Image‐Pro Plus 6.0; Media Cybernetics). At least 10 fields from each section were selected and evaluated. The immunostaining images were converted to grey scale, a linear combination between the average grey signal intensity and the relative area of positively stained cells was defined as the integrated optical density (IOD).

### 2.5 Male fertility assay

In vitro fertility (IVF) analysis was performed to assess male mice fertility. Normal female mice were super‐ovulated by i.p. injection of 10 i.u. pregnant mare serum gonadotrophin (PMSG) and human chorionic gonadotrophin (hCG) 48h apart. The oocytes were obtained at 14 h after injection of hCG and were cultured with overnight incubated HTF medium. About 35 oocytes could be obtained per female mouse, and each group was provided with 3 female mice. For sperm capacitation, mouse epididymal sperm wase collected in 200 μl C‐TYH medium for 0.5 h, which had pre‐balanced for 0.5 h, and all of these were done in a humidified 37℃, 5% CO_2_ incubator. About 10^6^ sperm/ml was transferred into HTF medium containing oocytes. 4–6 h after sperm‐egg incubation, embryos were washed in M2 medium for three times using appropriate diameter pasteur pipette to remove cumulus cells at the same time and placed in balanced KSOM medium with a paraffin overlay for following culture. Embryo development rate was defined as a percentage of the two‐cell embryos/the number of pronucleus formation oocyte. Blastocyte rate was defined as a percentage of the blastocytes/the number of the two‐cell embryos. For blastocyst quality, we adopted a three‐part scoring system according to Gardner’ report,^17^ which included blastocyst expansion (from early blastocyst with a less than half of the volume of the embryos to hatched blastocyst are graded from 1 to 6), inner cell mass (from tightly packed and many inner cells to very few cells are scored from A to C) and trophectoderm development (from many cells forming a cohesive epithelium to very few large cells are divided into grade A to C).

### 2.6 Statistical analysis

All data were presented as the mean ±standard deviation (SD) for three independent experiments. All statistical analyses were performed through GraphPad Prism 8 (GraphPad PrismA). The mean values were analysed by one‐way analysis of variance (ANOVA). A *p* value less than 0.05 was considered statistically significant.

## 3 RESULTS

### 3.1 PTX treatment impaired mice spermatogenesis and sperm quality

**FIGURE 1:**
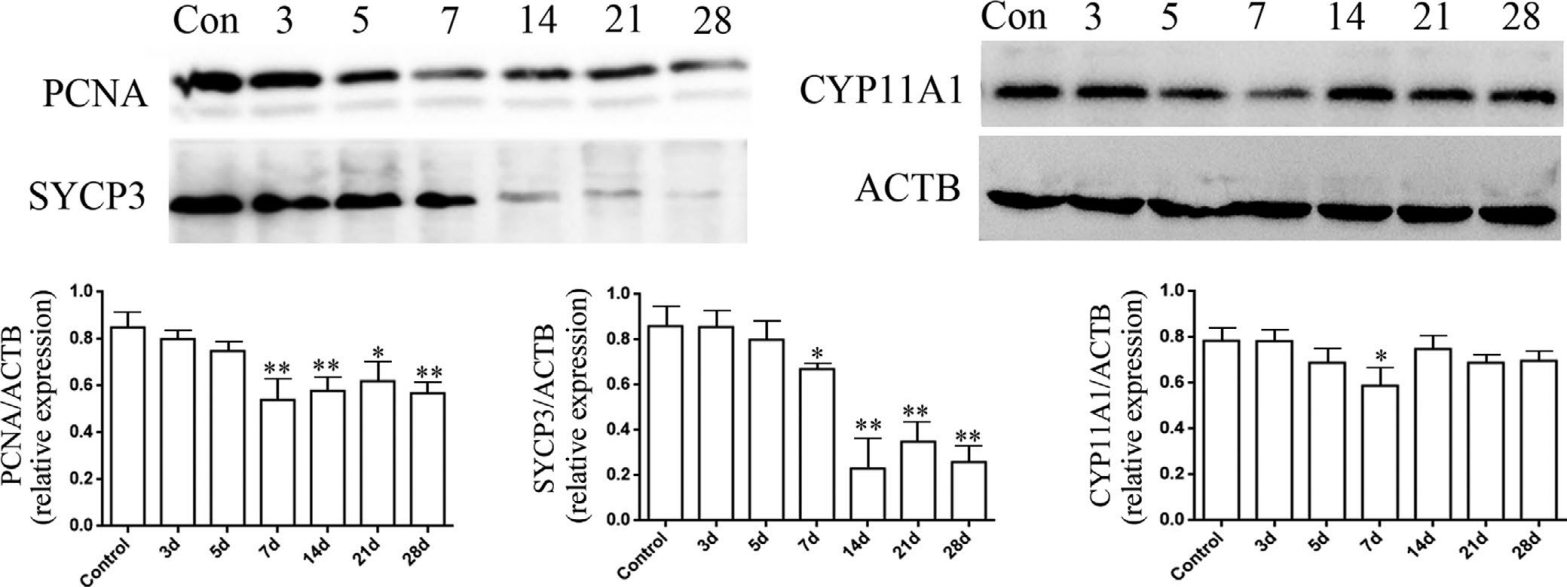
Western blot analysis of the expressions of PCNA, SYCP3 and CYP11A1 in mice testes at different days after PTX treatment. Samples were analysed by Western blot and repeated three times, followed by quantification through integrated optical density (IOD). The statistical analysis was performed by one‐way ANOVA; *p *< 0.05 was considered significant

Sperm parameters and expression of key proteins associated with spermatogenesis were analysed at Days 3, 5, 7, 14, 21 and 28 in PTX (10mg/kg body weight) group after PTX administration. Compared with control group, Western blot results indicated germ cell proliferation‐associated protein of PCNA, meiosis associated protein of SYCP3 and steroidogenesis associated protein of CYP11A1 were significantly decreased at 7 days after PTX treatment. The expressions of PCNA and SYCP3 decreased continuously from 7 days after PTX administration (Figure 1).

**FIGURE 2:**
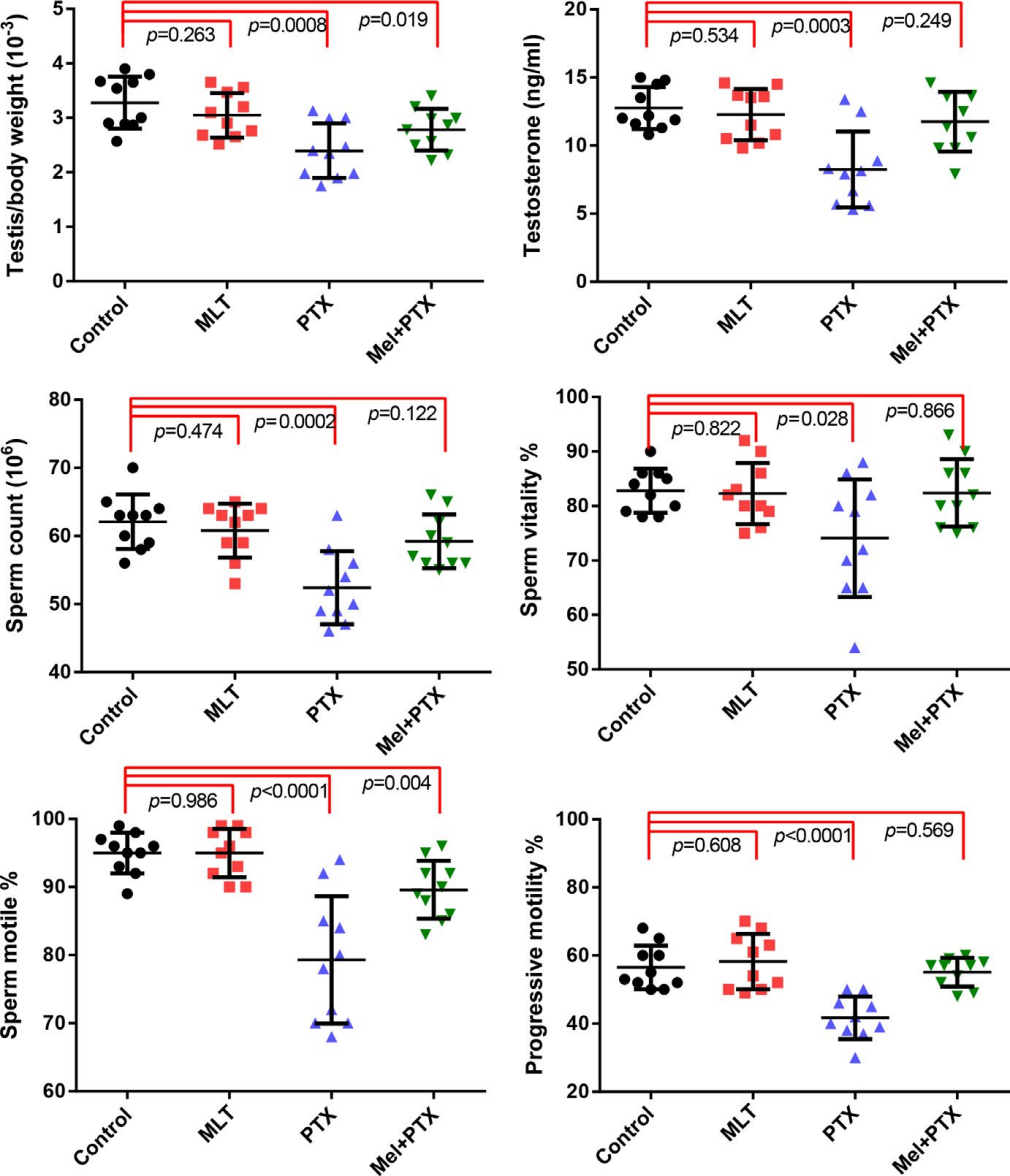
Characteristics of body weight, testosterone concentration, sperm motile and sperm motility in PTX‐treated mice at different time‐points after CP. Data of each group were obtained from ten mice and analysed by one‐way ANOVA; *p *< 0.05 was considered significant

We selected the time point of 14 days after PTX treatment to observe the ameliorating effect of MLT on the PTX‐induced reproductive toxicity. As shown in Figure 2, PTX treatment significantly reduced the levels of relative testis weight, serum testosterone, percentage of motile and living sperm, sperm counts and progressive motility rate. Administration of MLT obviously ameliorated the side effects of PTX, especially significantly increased serum testosterone levels and sperm progressive motility.

### 3.2 MLT ameliorated PTX‐induced impairment in mice testes and sperm

**FIGURE 3:**
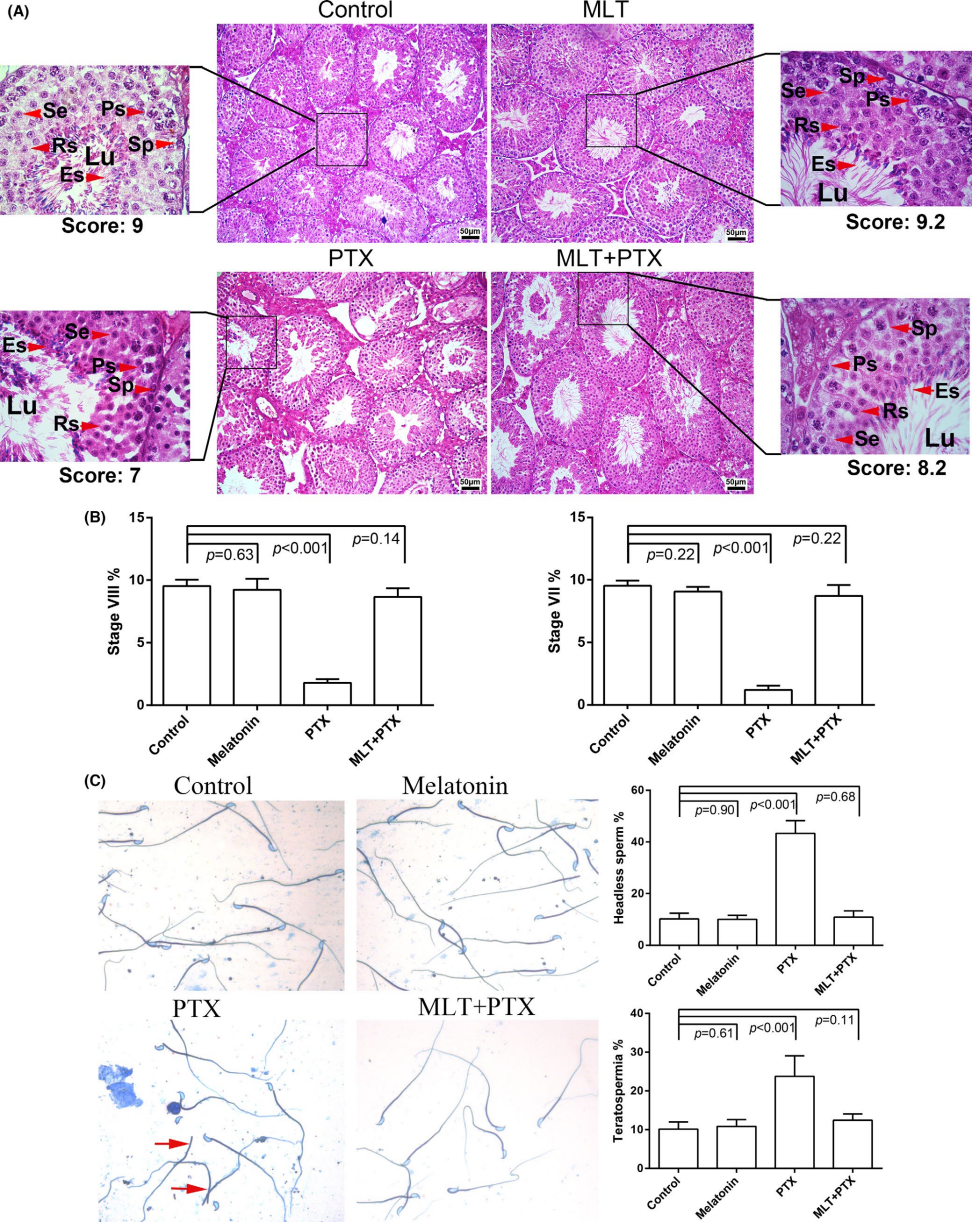
Morphological analysis of mice testes and sperm. Testes were obtained from control, MLT‐treated, PTX‐treated and MLT+PTX‐treated mice. A, The sections were stained by haematoxylin and eosin; B, Percentage of stage VII and VIII tubules were calculated and statistically analysed; C, Sperm were stained by Coomassius. Melatonin, MLT; Paclitaxel, PTX; Sp, spermatogonia; Ps, pachytene spermatocyte; Se, secondary spermatocyte; Rs, round spermatid; Es, elongated spermatids; The data were analysed through one‐way ANOVA, *p *< 0.05 was considered as significant. Each bar represents 50 μm

As shown in Figure 3A, control and MLT‐treated mice showed normal testis morphology, while PTX changed testicular morphology clearly, including thinning of germ cell layers in seminiferous tubules, vacuoles and loose interstitial structures. A score evaluation showed that PTX treatment led to testiclular atrophy (Supplementary Table 2). PTX also significantly reduced the numbers of stage VII and VIII seminiferous tubules, which indicated the obvious influence on meiosis, while MLT administration noticeably ameliorated the reproductive toxicity induced by PTX. Morphological analysis of sperm revealed that PTX treatment markedly increased the proportion of headless sperm (42%) and teratospermia sperm (25%). There were no significant differences present in sperm abnormality rate between the PTX+MLT group and normal group (Figure 3B).

### 3.3 MLT increased expressions of proteins associated with spermatogenesis that were reduced by PTX

**FIGURE 4:**
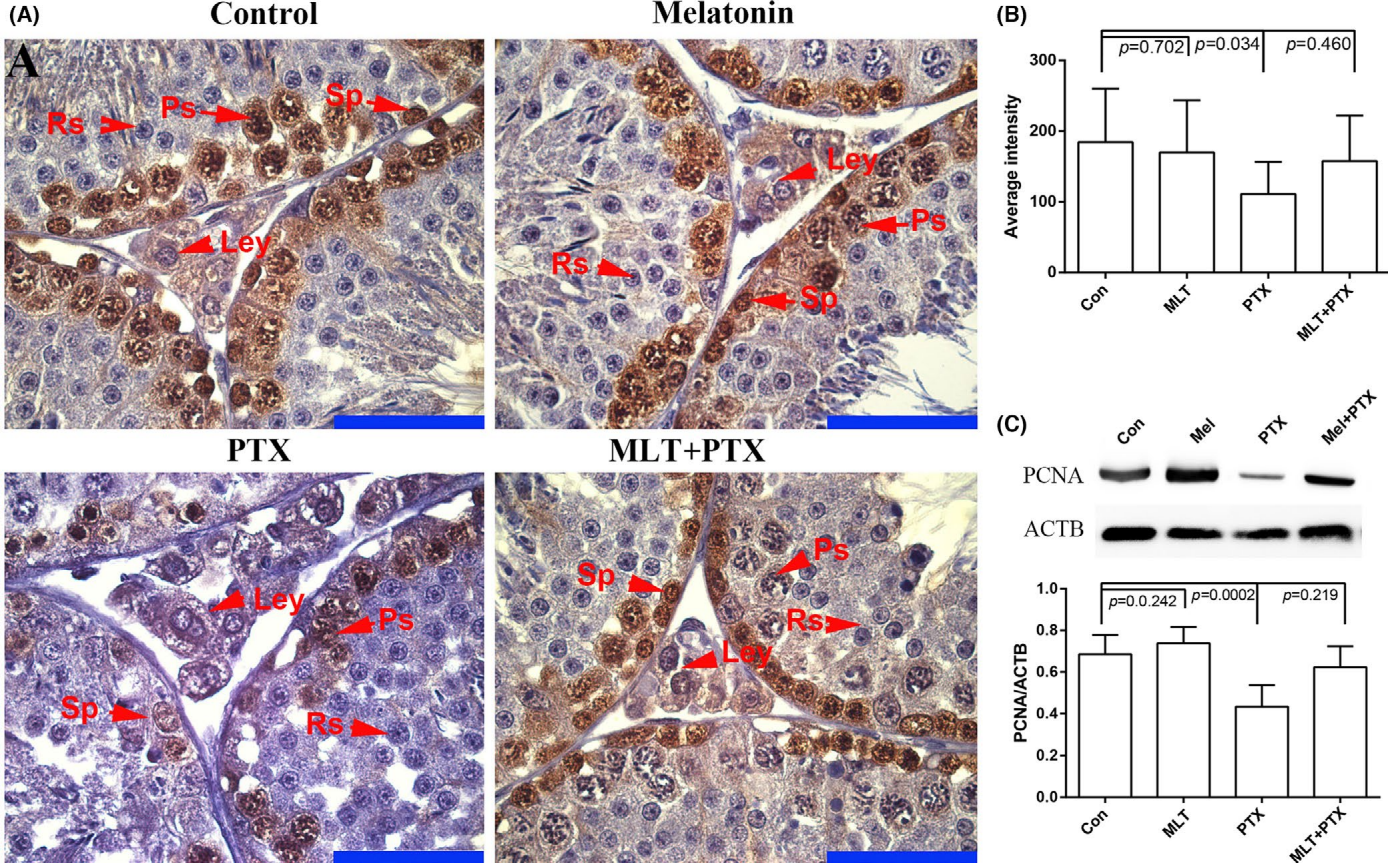
Expression of PCNA in in control and PTX‐treated mice testes Testes were obtained from control, MLT‐treated, PTX‐treated and MLT+PTX‐treated mice. The sections were stained by haematoxylin and eosin. Mel, MLT; PTX, PTX. A, immunostaining of PCNA in testes; B, quantification expression of PCNA in testes; C, Western blot analysis of PCNA in testes in triple replications. The data were analysed through one‐way ANOVA, *p *< 0.05 was considered as significant. Each bar represents 50 μm

To study the effect of MLT on the proliferation of germ cells, PCNA expression in the testis was detected by immunohistochemistry and Western blot. PCNA, mainly expressed in spermatogonia and spermatocyte cells, showed a decreased expression in PTX‐treated mice testes and increased expression after MLT administration (Figure 4). Meanwhile, Western blot analysis also presented a similar trend of increasing PCNA expression by MLT administration in PTX‐treated mice, but no significant differences were observed between control and single MLT group.

**FIGURE 5:**
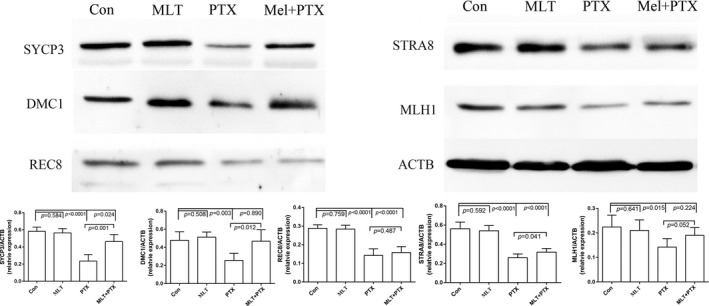
Expressions of key spermatogenesis‐related in control and PTX‐treated mice testes. Samples were analysed by Western blot and repeated three times, followed by quantification through their IODs. The statistical analysis was performed by one‐way ANOVA; *p *< 0.05 was considered significant. Mel, MLT; PTX, PTX

Significantly decreased expression of meiosis‐related protein of SYCP3 in PTX‐treated mice testes indicated a side effect on meiosis (Figure 1). Meiosis‐related proteins of SYCP3, DMC1, REC8, STRA8 and MLH1 were detected between MLT and MLT plus PTX‐treated mice testes by Western blot. The results showed PTX induced noticeably decreased expressions of meiosis‐related proteins, while MLT remarkably improved the decreased expressions of these proteins, especially of SYCP3, DMC1 and MLH1 (Figure 5).

### 3.4 MLT improved expressions of fertility‐related proteins of HSPA2 and HSPA4L in PTX‐treated mice testes

**FIGURE 6:**
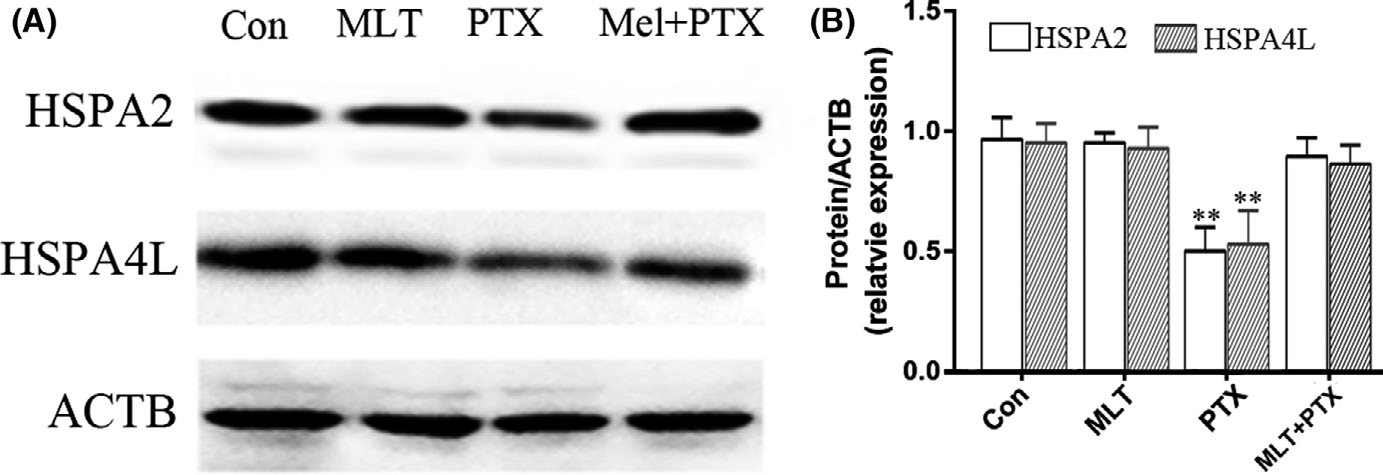
Expressions of HSPA2 and HSPA4L in control and PTX‐treated mice testes. Mel, MLT; PTX, PTX. The data were analysed through one‐way ANOVA; **, *p *< 0.01 was considered as significant

Expressions of HSPA2 and HSPA4L, known as fertility‐related proteins, were detected in the testes of PTX‐treated and MLT+PTX‐treated mice to determine the impact of MLT administration on their expressions. MLT significantly ameliorated decreased tendency of HSPA2 and HSPA4L induced by PTX (Figure 6).

### 3.5 MLT rescued male fertility in PTX plus MLT‐treated mice

**FIGURE 7:**
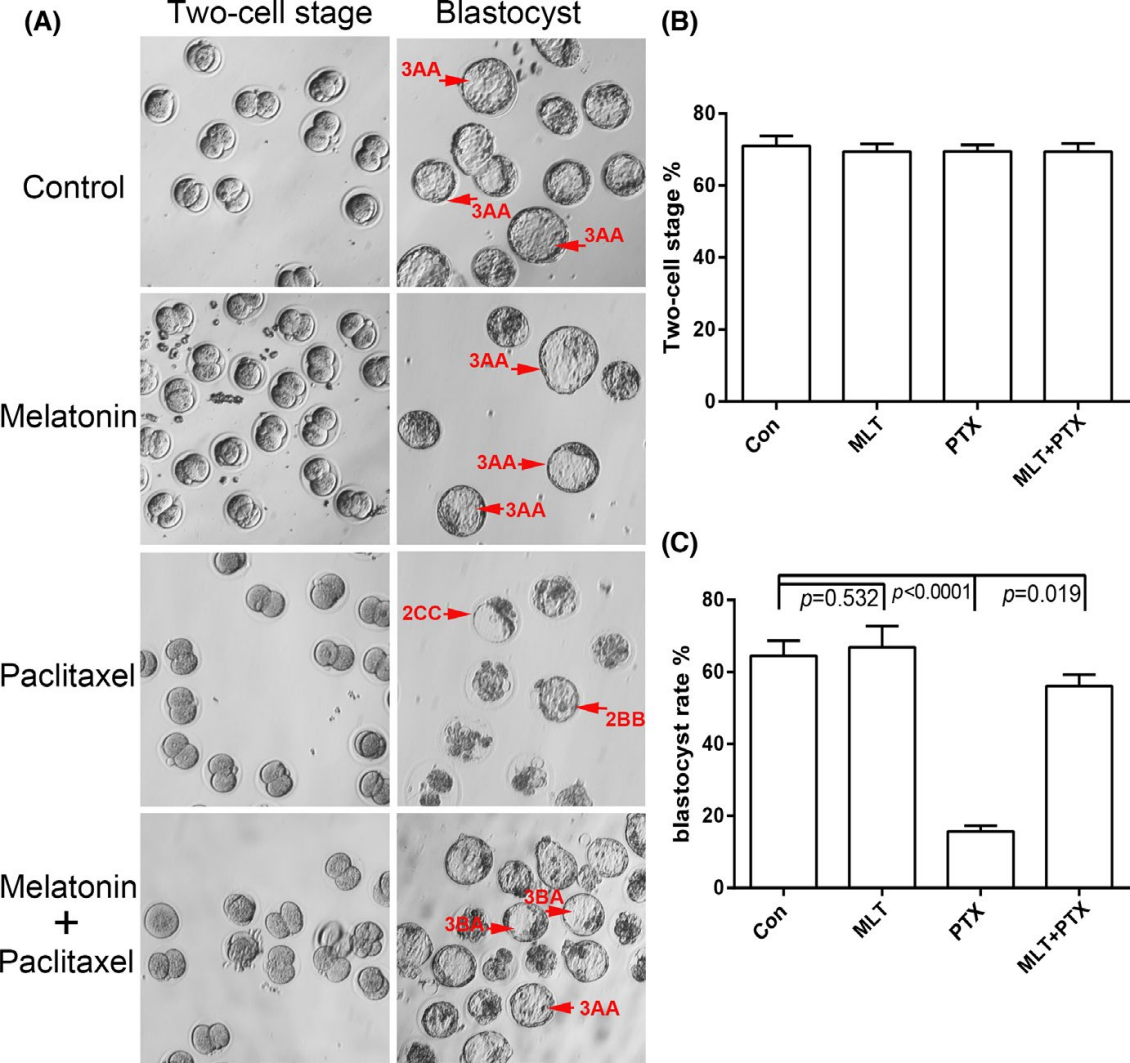
Characteristics of male fertility of MLT and PTX‐treated mice by IVF analysis A, Representative image of two‐cell embryos and blastocysts in different groups; B, Statistical analysis of two‐cell stage rate, which was defined as a percentage of the two‐cell embryos/ the number of pronucleus formation oocytes; C. Statistical analysis of blastocyte rate, which as defined as a percentage of the blastocytes/ the number of the two‐cell embryos. *p *< 0.05 was considered as the significant

We analysed the male fertility ability through IVF, mainly including the two‐cell development and blastocyst formation. The results showed that PTX had no effects on the development of two cells, but significantly reduced the quantity and quality of blastocyst, which could be significantly improved by MLT administration (Figure 7). Top score blastocysts (≧3AA) were very common in control or MLT groups, while 2CC or 2BB blastocysts were obtained in PTX group. MLT administration changed the quality partially and 3AA or 3BA blastocysts could be found in MLT+PTX group. Since both the quality and quantity of blastocysts were important predictors of pregnancy outcome, MLT could rescue male fertility destroyed by PTX.

## 4 DISCUSSION

In this study, we established a PTX‐treated mouse model to study the effects of PTX on spermatogenesis and male fertility, providing important information for further understand of male reproduction affected by chemotherapy drugs. Meanwhile, the protective effects of MLT on PTX‐induced reproductive damage were discussed in spermatogenesis and sperm function.

It was reported that PTX might induce spermatogenesis disorder, but the molecular mechanism was unclear. Key molecules related to germ cell proliferation, meiosis and steroidogenesis were first detected, and these markers could assess the effects of PTX on male fertility and identify the key spermatogenesis processes mostly affected. The results showed that PTX could affect the expression of PCNA and SYCP3 in testis. PCNA is an important biomarker related with spermatogonia and spermatocytes proliferation,^18^ and SYCP3 is a well‐known biomarker associated with meiotic spermatocytes.^19^ It suggested that PTX might affect the spermatogenesis process by disturbing the proliferation of germ cells and the meiosis process, resulting in diminished sperm quantity and quality. This effect began to appear obviously at 7 days after the PTX administration and significantly affected meiosis process from the 14th day. Thus, we selected samples at 14 days after PTX administration as the main research object for indepth study. PTX significantly reduced the testis weight, motile sperm and sperm motility. Morphologically, the germ cells in the seminiferous tubules of the PTX‐treated mice were significantly reduced, with obviously decreased stage VII and VIII tubules, which corresponded to the loss of testis weight and sperm counts. Sperm count reduction is an important indicator of male infertility.^20^, ^21^ Motile sperm and motility are important parameters of semen quality, and male infertility is directly related to quality and quantity of sperm within the semen^22^; so, PTX caused obvious testicular dysfunction via affecting sperm count and quality. Because PLT can increase production of ROS and mammalian spermatozoa are vulnerable to ROS,^23^ ROS was considered to be one of the important factors leading to these injuries.

As well known, MLT, which is involved in the proliferation of spermatogenic cells,^24^ is an important antioxidant that can affect sperm quality by reducing oxidative stress or other molecular mechanisms. Although previous studies have reported the beneficial effects of MLT on testicular cells under pathological conditions, it is unclear whether MLT can protect the testis against PTX‐induced testicular damage. In this study, the protective effect of MLT on spermatogenesis and sperm function in PTX‐treated mice was studied, which provided an important basis for further study of its molecular function and molecular mechanism. We found that MLT could significantly improve the testicular morphology changed by PTX and protect the sperm function, especially increasing the numbers of the VII and VIII stage germ cells, which plays an important role in the maintenance of spermatogenesis process and subsequent sperm function. Consistent with morphological changes, MLT attenuated PTX‐induced sperm quality degradation, including sperm motile ratio and sperm motility. However, no significant phenotypic improvements were observed in mice treated with MLT alone compared with normal control mice, suggesting that MLT might ameliorate spermatogenesis and sperm quality‐related damage by mitigating the side effects of PTX.

We focused on germ cell proliferation, meiosis and sperm quality to conduct indepth verification in order to provide important information for further research on the molecular mechanism of MLT's protective effect. Combining all the results, we proposed that MLT ameliorated the decrease of each molecular index caused by PTX because of its antioxidant characteristic and the subsequent morphological and molecular level changes caused by oxidative damage were the main factors in the process of PTX's influence on spermatogenesis. Furthermore, MLT also had protection effect on sperm quality. HSPA2 and HSPA4L, important fertility‐related proteins and closely related to sperm quality,^25^, ^26^ were significantly improved by MLT in PTX‐induced mice. PTX led to the decrease of sperm quality, which was not only reflected in the parameters related to sperm quality, but also in the embryo development ability. In vitro fertilization, blastocyst formation rate and quality in the PTX group were significantly reduced when compared with control group, while MLT obviously prevented this decrease, and the result of MLT‐treated alone blastocysts was similar to that in the control group. PTX had little effect on formation of two cells, but mainly on blastocyst formation. It has been reported DNA damage of sperm had a very clear relationship with sperm concentration, motility and morphology, while it had no relationship with either fertilization rates or mean embryo score on Day 2 or 3.^27^ Inherent defect in the sperm had a negative influence on blastocyst development,^28^ while paternal genome was activated after 2‐cell stage, until which point zygotic genome activation took place.^29^ It suggested that PTX‐induced abnormal spermatogenesis was an important source factor affecting sperm quality, and MLT demonstrated a reversal of this effect.

In conclusion, the reproductive toxicity of PTX and the protective effect of MLT were studied in two aspects of spermatogenesis and sperm quality. MLT protected the spermatogenesis process via improving the proliferation of germ cells and meiosis process influenced by PTX. Meanwhile, MLT significantly protected the sperm quality to maintain the male fertility potential. The molecular mechanism of MLT involved in spermatogenesis and sperm quality protection was warranted to be further study.

## CONFLICT OF INTEREST

The authors declare that there is no conflict of interest.

## AUTHOR CONTRIBUTIONS

**ZhiXin Wang:** Data curation (equal); Methodology (equal); Validation (equal). **Zi Teng:** Data curation (equal); Methodology (equal). **Zelin Wang:** Investigation (supporting); Methodology (supporting); Validation (supporting). **Zhan Song:** Investigation (supporting); Methodology (supporting); Validation (supporting). **Peng Zhu:** Investigation (supporting); Methodology (supporting); Validation (supporting). **Ning Li:** Investigation (supporting); Methodology (supporting); Validation (supporting). **Yusheng Zhang:** Investigation (supporting); Methodology (supporting). **XueXia Liu:** Conceptualization (equal); Funding acquisition (equal); Supervision (equal); Validation (equal); Writing – original draft (equal); Writing – review & editing (equal). **FuJun Liu:** Conceptualization (equal); Funding acquisition (equal); Investigation (equal); Supervision (equal); Writing – original draft (equal); Writing – review & editing (equal).

## Supporting information

Table S1

Table S2

## Data Availability

Data sharing is not applicable to this article as no new data were created or analysed in this study.
